# Predicting Intensive Care Unit Admission in COVID-19-Infected Pregnant Women Using Machine Learning

**DOI:** 10.3390/jcm13247705

**Published:** 2024-12-17

**Authors:** Azamat Mukhamediya, Iliyar Arupzhanov, Amin Zollanvari, Saule Zhumambayeva, Kamalzhan Nadyrov, Zaituna Khamidullina, Karina Tazhibayeva, Aigul Myrzabekova, Kulyash K. Jaxalykova, Milan Terzic, Gauri Bapayeva, Saltanat Kulbayeva, Gulzhan Narkenovna Abuova, Baktigali Aubayevich Erezhepov, Asselzhan Sarbalina, Aigerim Sipenova, Kymbat Mukhtarova, Ghazal Ghahramany, Antonio Sarria-Santamera

**Affiliations:** 1 Department of Electrical and Computer Engineering, School of Engineering and Digital Sciences, Nazarbayev University, Astana 010000, Kazakhstan; 2 Astana Medical University, Astana 010000, Kazakhstan; 3 Department of Surgery, School of Medicine, Nazarbayev University, Astana 010000, Kazakhstan; 4 Clinical Academic Department of Women’s Health, Corporate Fund “University Medical Center”, Astana 010000, Kazakhstan; 5 Department of Obstetrics and Gynecology, South Kazakhstan Medical Academy, Shymkent 160000, Kazakhstan; 6 Shymkent City Infectious Disease Hospital, Shymkent 160000, Kazakhstan; 7 University of Oxford, Oxford OX3 9DU, UK; 8 Department of Biomedical Sciences, School of Medicine, Nazarbayev University, Astana 010000, Kazakhstan

**Keywords:** COVID-19, intensive care unit admission, pregnancy, machine learning, feature importance

## Abstract

**Background**: The rapid onset of COVID-19 placed immense strain on many already overstretched healthcare systems. The unique physiological changes in pregnancy, amplified by the complex effects of COVID-19 in pregnant women, rendered prioritization of infected expectant mothers more challenging. This work aims to use state-of-the-art machine learning techniques to predict whether a COVID-19-infected pregnant woman will be admitted to ICU (Intensive Care Unit). **Methods**: A retrospective study using data from COVID-19-infected women admitted to one hospital in Astana and one in Shymkent, Kazakhstan, from May to July 2021. The developed machine learning platform implements and compares the performance of eight binary classifiers, including Gaussian naïve Bayes, K-nearest neighbors, logistic regression with *L_2_* regularization, random forest, AdaBoost, gradient boosting, eXtreme gradient boosting, and linear discriminant analysis. **Results**: Data from 1292 pregnant women with COVID-19 were analyzed. Of them, 10.4% were admitted to ICU. Logistic regression with *L_2_* regularization achieved the highest *F*_1_-score during the model selection phase while achieving an AUC of 0.84 on the test set during the evaluation stage. Furthermore, the feature importance analysis conducted by calculating Shapley Additive Explanation values points to leucocyte counts, C-reactive protein, pregnancy week, and eGFR and hemoglobin as the most important features for predicting ICU admission. **Conclusions**: The predictive model obtained here may be an efficient support tool for prioritizing care of COVID-19-infected pregnant women in clinical practice.

## 1. Introduction

The rapid onset of COVID-19 placed immense strain on many already overstretched healthcare systems. As a result of the acute shortage of hospital beds and medical staff during the pandemic, various stringent patient triage protocols were introduced. The unique physiological changes during pregnancy present challenges in understanding the full scope and effects of the complexity of COVID-19 infection on pregnant women [1], rendering the clinical decision-making in these patients and risk-prioritization of expectant mothers even more challenging.

The disruption of SARS-CoV-2 not only significantly impacted societies, economies, and mental well-being but also translated into the need to accurately estimate patient prognosis based on patient-specific risk. Identifying patients at high risk of complications is critical in moments of high caseloads. In the gestational period, the presentation of COVID-19 can vary significantly, encompassing asymptomatic cases, mild respiratory symptoms that require minimal supportive treatment, and severe cases leading to hospitalization with multi-organ failure and death [2]. Given the significant changes in the immune, circulatory, respiratory, and hormonal systems in addition to specific problems that appear during this period, such as preeclampsia or gestational diabetes, the full understanding of the COVID-19 impacts on pregnant women is yet to be clarified [3]. Moreover, research exploring the influence of different pregnancy trimesters on the disease’s clinical progression and complications is scarce [4].

One of the critical factors during the pandemic was prioritizing patients in need of intensive care to avoid unnecessary consumption of medical resources on low and moderate-risk patients [5]. The sudden COVID-19 outbreak intensified the shortage of hospital beds, critical care equipment, and medical professionals [6]. Proper triage systems to predict the clinical course of patients become essential for efficient management of limited medical resources, including intensive care [7].

Machine learning may classify severity and assess prognosis for COVID-19 patients across a variety of routinely collected laboratory tests and clinical data [8]. In this regard, machine learning can be viewed as a useful technique for supporting caregivers in medical decision-making, and it has been utilized in multiple COVID-19 studies to construct models that predict the severity of SARS-CoV-2 patients [9,10]. Moreover, the constructed predictive models can be used to accurately assess the risk of the outcome and will allow for individualized preventive measures in clinical settings. As a result, in this study, we aim to (i) develop a machine learning model that can predict the risk of ICU admission in COVID-19-infected pregnant women based on clinical data routinely collected during hospitalization and (ii) shed light on major factors associated with the higher risk of ICU admission for pregnant women with COVID-19 infection.

## 2. Materials and Methods

### 2.1. Study Population

This retrospective study was conducted using de-identified data from medical records of pregnant women with COVID-19 admitted at Astana Perinatal Hospital and at the Department of Obstetrics and Gynecology South Kazakhstan Medical Academy in Shymkent from 1 May 2021 to 14 July 2021. Inclusion criteria were pregnant women who were admitted to any of the previously mentioned hospitals in the period from 1 May 2021 to 14 July 2021 with SARS-CoV-2 infection confirmed through real-time polymerase chain reaction (RT-PCR). The dataset includes 46 variables, which broadly cover information about days of admission after the onset of the symptoms, length of hospital stay, obstetric history, laboratory tests, clinical symptoms, and severity of COVID-19, comorbidities, and complications (see Table 1 for detailed information about the variables). The comorbid disorders were presented as categorical variables encoded as “yes” and “no” subgroups. ICU admission was chosen as an outcome variable for prediction. The dataset is imbalanced, with the ratio of those admitted to ICU to those who are not admitted at 110:1058. Note that we did not consider using any methods to address the class imbalance directly. Instead, we used an alternative strategy to handle the class imbalance by considering the decision-making threshold as a hyperparameter and tuning it in the model selection stage.

Ethical approval was obtained from Nazarbayev University Institutional Review Ethics Committee (NU-IREC) #745/12062023. The data were extracted from electronic medical records by practicing clinicians from the hospitals and provided de-identified for conducting these analyses. All methods were carried out in accordance with the “Reporting of studies conducted using observational routinely collected health data” (STROBE) guidelines.

### 2.2. Data Pre-Processing

Several pre-processing steps were employed to prepare the dataset for the analysis. Initially, categorical variables were transformed using one-hot encoding. The dataset was randomly split into training and test sets. Missing feature values in the training and test sets were imputed based on the median of those values in the training data to prevent any data leakage between the training and test sets. The training set was normalized by applying standardization, whereas the test set was normalized based on the statistics obtained from the training set.

### 2.3. Model Selection

**Figure 1:**
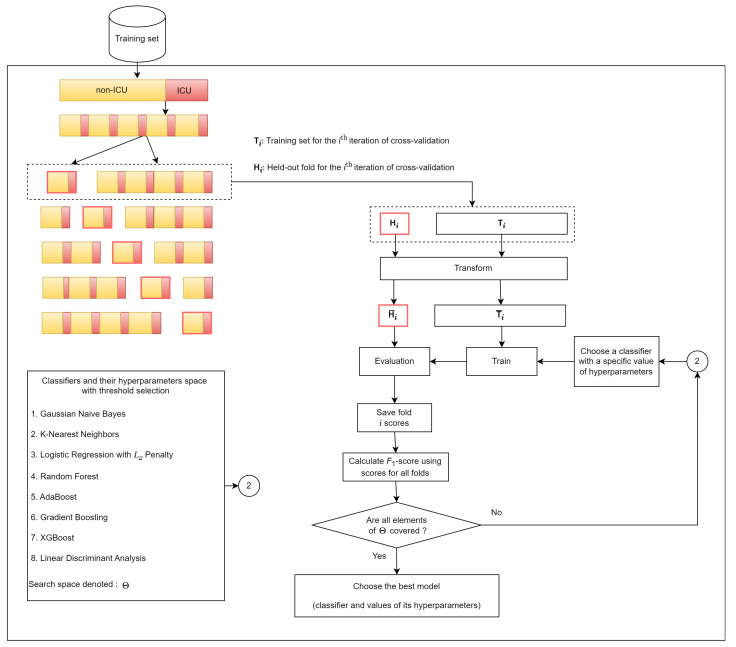
A schematic diagram of the model selection procedure.

Eight classifiers were used: Gaussian Naïve Bayes (GNB) [11]; K-Nearest Neighbors (KNN) [11]; Logistic Regression with *L_2_* regularization (LR) [12]; Random Forest (RF) [13]; AdaBoost (ADB) [14]; Gradient Boost (GB) [15]; eXtreme Gradient Boost (XGB) [16]; and Linear Discriminant Analysis (LDA) [17]. The rationale behind selecting these predictive models is as follows: (i) the selected models represent five well-known groups: ensemble; Gaussian process; nearest neighbor; linear models; and discriminant analysis, and (ii) these models have been widely utilized in previous studies to predict ICU admission [18,19,20] and COVID-19 severity risk [5,8,9]. The best predictive model was selected according to the highest *F*_1_-score that was estimated using a stratified 5-fold cross-validation strategy applied to the training set. Figure 1 presents the schematic diagram of the model selection procedure.

It is worth noting that the decision-making threshold was considered a hyperparameter and tuned in the model selection stage. Particularly, a classifier outputs the predicted class label for a given observation by comparing its assigned score with a predetermined threshold value. In this work, instead of using a default value (often 0 or 0.5), we treated the threshold value as an essential hyperparameter and optimized it along with other hyperparameters as a part of model selection. The hyperparameter space for threshold values includes values from 0 to 1 with a step size of 0.01. The classifier-specific hyperparameter spaces are detailed in Table 2.

### 2.4. Model Evaluation

The selected and trained predictive model was evaluated on the test set based on the following performance metrics: area under the curve (AUC); precision; sensitivity (recall); specificity; *F*_1_-score; and geometric mean of sensitivity and specificity (G-mean). These metrics are defined as follows:

$$precision=\frac{TP}{TP+FP},$$

$$specificity=\frac{TN}{TN+FP},$$

$$sensitivity=\frac{TP}{TP+FN},$$

$$F_{1}–score=\frac{2\times precision\times recall}{precision+recall},$$

$$G–mean=\sqrt{specificity\times sensitivity}$$

where TP, FP, TN, and FN are the number of true positives, false positives, true negatives, and false negatives, respectively.

### 2.5. SHAP Analysis

Shapley Additive exPlanations (SHAP) analysis (version 0.44.1) [21] was used to examine the effect of each feature on the prediction of the classifier and assess the importance of the feature in predicting admission to the ICU.

### 2.6. Software and Packages

The entire computational pipeline was implemented in Python (version 3.9.7) using the scikit-learn library. The computations were performed using the MacOS 13.1 (Ventura) operation system with Apple chip M1 and 8GB of RAM (Cupertino, CA, USA).

## 3. Results

### 3.1. Data Description

In this study, the initial database included 1292 cases collected from Astana Perinatal Hospital and the Department of Obstetrics and Gynecology of South Kazakhstan Medical Academy Shymkent. After eliminating those with incomplete data, 1168 pregnant women with COVID-19 were available for analysis (see Section 2.1). Specifically, the samples that contained missing values in more than 20% of their features were removed. Missing values in each feature were imputed by the median of feature values across the entire sample. The data include 22 binary, 2 categorical, and 22 numeric features (see Table S1). The training and test sets were obtained by randomly dividing entire data into two disjoint sets with a splitting ratio of 70:30. The smaller subset was used as a test set, and the larger subset was used for training purposes. Stratification was used to keep the proportion of classes that appear in the training and test splits the same as the full dataset. The training set was used to obtain the best predictive model, while the test set was used for the evaluation of the obtained model. The main characteristics of the data are shown in Table 1. 87.3% of cases were in the third trimester, and 10.4% required ICU admission.

### 3.2. Predictive Performance

**Figure 2:**
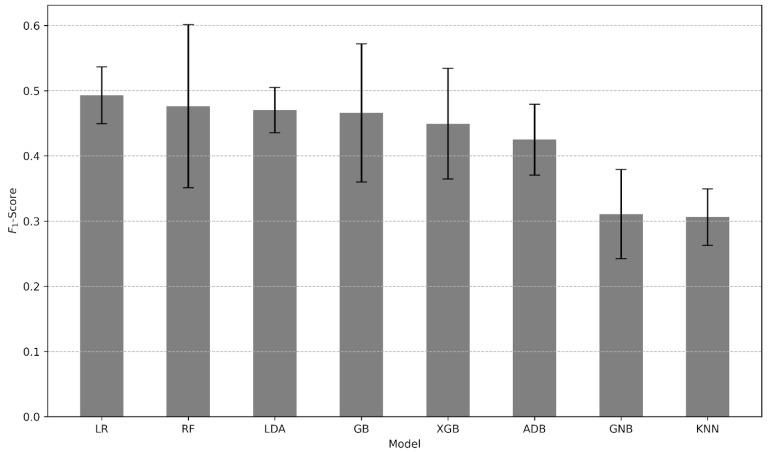
Estimated *F*_1_-scores mean ± standard deviation using 5-fold cross-validation obtained on the training set.

As a result of model selection, the highest *F*_1_-score of 0.493 ± 0.0434 was achieved by the LR model (see Figure 2). In particular, the best model is obtained based on a pipeline consisting of the hyperparameter tuning, including the decision-making threshold estimation. In Table 3, the estimate of *F*_1_-scores, the estimated threshold value, and other estimated hyperparameters of each classifier are presented. Ultimately, the LR model with selected parameters and decision-making threshold value was evaluated using the test set. Table 4 presents the test-set performance metrics estimated using the best predictive LR model. The test-set confusion matrix for the LR classifier is presented in Table 5. The confusion matrix reflects a positive predictive value of 86%. Values of 87.3% of ICU admissions were in women in the third trimester. Cases with ICU admission were older, with more pregnancies and deliveries. They had lower saturation, hemoglobin, and lymphocytes, as well as more elevated leucocytes, neutrophils, platelets, and APTT. Preeclampsia, hypertension, hyperglycemia, and gestational diabetes were also frequent among these women.

### 3.3. Impact Direction and Importance of Each Feature

**Figure 3:**
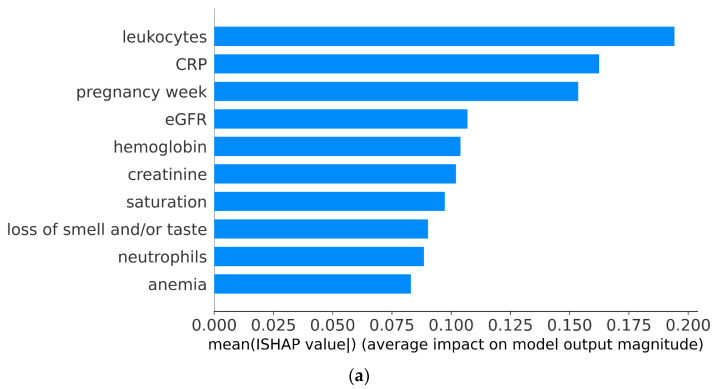

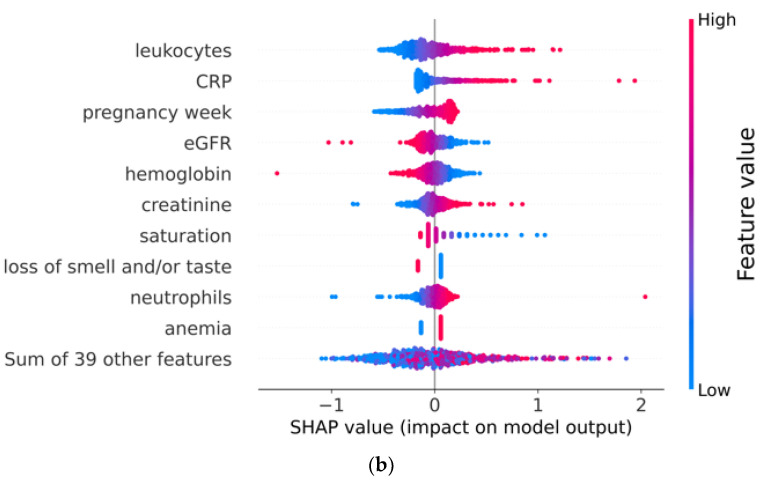
(**a**) Bar plot of top 10 features (from highest to lowest importance) based on mean SHAP value. (**b**) Beeswarm plot of top 10 features (from highest to lowest importance) based on SHAP value.

We utilized a SHAP analysis to assess the importance of each feature on ICU admission prediction using the trained LR classifier. Figure 3a,b depicts the bar and beeswarm plot of the ten most important features according to SHAP values. In particular, the bar plot shows the ranking of the features in terms of their importance. In this case, we can see that leukocytes, CRP, and pregnancy week are the most important features on average. On the other hand, the beeswarm plot is designed to display an information-dense summary of how the top features in a dataset impact the model’s output. Each instance of the given explanation is represented by a single dot on each feature. Each position of the dot is determined by the SHAP value of that feature. Dots “pile up” along each feature row so that the width at a certain point represents the density. The color code indicates the protective (blue) or higher risk (red) of admission to the ICU. For example, the high value of leukocytes yields positive SHAP values, meaning the direct dependence between leukocytes and a higher risk of admission to the ICU. At the same time, the high value of hemoglobin decreases the risk of ICU admission as it corresponds to negative SHAP values.

## 4. Discussion

We collected data from 1168 patients to construct predictive models of adverse outcomes related to SARS-CoV-2 infection in pregnancy. The constructed model aims to conduct higher-level risk stratification to provide efficient decision-making and advance personalized medicine upon obtaining a positive SARS-CoV2 test in pregnant patients. Our machine learning-based ICU admission predictive model can potentially be used as an auxiliary tool for supporting caregivers in medical decision-making. To construct and compare our predictive models, we leveraged a threshold-dependent metric of performance (*F_1_*-score) to identify the best model.

As seen in Table 4, the best predictive model (LR) demonstrates a predictive performance in the range of 0.8–0.9 in terms of AUC. As per objective metrics of diagnostic tests, an estimated AUC in the range of 0.8–0.9 is generally considered a “good” predictive capacity for the test [22]. At the same time, LR correctly predicted 283 true negatives and 21 true positives, which correspond to a specificity of 0.896 and a sensitivity of 0.600. In addition, our model can predict cases with a low risk of developing adverse outcomes requiring ICU admission during hospitalization with a rate of 283/(283 + 33) = 0.89.

Our model achieved a moderate *F*_1_-score of 0.472 with a 0.600 recall and 0.389 precision. That is to say, the model shows a satisfactory ability to identify positive cases but has lower reliability in its positive predictions. This is a natural outcome of selecting a low decision-making threshold value (the best predictive model, LR, has a threshold value of 0.18; see Table 3). It is worth noting that having high recall might be beneficial in cases where missing positives is more costly, for example, in medical diagnoses. Another possible cause of the moderate *F*_1_-score is class imbalance. Recall that our dataset has a skewed class distribution (see Section 2.1).

Several studies have employed a machine learning approach to predict ICU admission for COVID-19-infected patients [18,19,20]. For example, Karimi et al. [18] compared different machine learning models to predict the requirements for ICU admission in COVID-19 patients. In their work, they considered six models: Support Vector Machine (SVM); Naïve Bayes; LRR; light GBM; decision tree (DT); and KNN. Among them, the best predictive model, Naïve Bayes, achieved 0.71 AUC and 0.40 *F_1_*-score. Famiglini et al. [19] used four models, multi-layer perceptron, DT, SVM, and XGB, to predict ICU admission for COVID-19 patients within any of the next five days. The best predictive model, SVM, showed 0.85 AUC and 0.54 *F_1_*-score. Cheng et al. [20] used the RF model as a risk prioritization tool to predict COVID-19 ICU admission within 24 h. Their model achieved 0.80 AUC and 0.18 *F_1_*-score.

During the COVID-19 pandemic, the perinatal centers in Astana and Shymkent were repurposed as infectious disease hospitals. All pregnant women with a positive result for coronavirus infection in these cities were admitted to this hospital, generating the need for valid triage systems to determine the most efficient process of care for each patient within the consistently limited resources, like intensive care unit beds.

The overall proportion of ICU admissions was 10.4%, as reported by some authors [23], although some authors have reported lower values [24]. Both differences in clinical characteristics of the patients included in these analyses, as well as the period of the pandemic and circulating variants and specific healthcare-related issues like availability of ICU beds and vaccination status, may explain these differences.

SHAP analysis is an effective technique to quantify the importance of features [25]. The results of our SHAP analysis demonstrate that the most important features are leukocyte and C-reactive protein. Leukocytosis is a hematological parameter associated with more severe COVID-19 infection in pregnant women, along with neutrophilia, lymphopenia, and elevated platelet counts [26,27]. While the precise pathophysiological explanation of these hematological abnormalities in COVID-19 patients is still unclear, changes in the number of one or more blood cells have often been reported frequently in patients infected with SARS-CoV-2 [28]. Leukocytosis and neutrophilia are inflammatory responses associated with disease severity and bad outcomes. Although neutrophilia may be more specific to severe disease than leukocytosis [29], leukocyte counts have been observed more frequently, compared with neutrophil ones, in more severe cases of COVID-19-infected pregnant women [30]. C-reactive protein (CRP) is another relevant factor associated with the risk of ICU admissions for pregnant women infected with COVID-19. CRP is recognized as a robust marker of acute systemic inflammation and severe infection [31]. Elevated CRP concentrations are associated with COVID-19 severity [32] but are also typically reported in severe viral infections, including H1N1 influenza pneumonia [33,34]. Elevated CRP levels are associated with severe COVID-19 cases in pregnancy [35] and have been linked with ICU admission, preterm labor, and poor maternal outcomes [36], as they reflect underlying inflammatory responses that are heightened in severe infections [37]. We note that the “importance” of the feature indicates the “association” between the feature and the output of the model.

Lymphopenia is another indicator of severe COVID-19 infection. In pregnant women, lymphopenia often signals an impaired immune response, placing these patients at higher risk for severe infection and complications, including ICU admission and preterm delivery. Neutrophil-to-lymphocyte ratios are valuable for detecting potentially critical and fatal cases of COVID-19 [38].

In terms of platelet counts, in cases admitted to ICU, even though they showed higher levels, they still remained in the normal range. Mild thrombocytopenia has been reported frequently in COVID-19 infection [39] as well as rebound thrombocytosis [40]; however, none of these factors were detected as major contributing elements associated with ICU admission of COVID-19-infected pregnant women by our predictive models.

Anemia and low hemoglobin levels were also associated with a higher risk of severe infection requiring ICU [41]. From an etiological standpoint, thrombosis, hemorrhage, and autoimmunity have been suggested for this association. During pregnancy, hemoglobin concentration is decreased. This is due to higher blood volume, which supplies oxygen and nutrients to the uterus, placenta, and other organs. Iron deficiency may also occur in pregnant women due to an increased need for iron, leading to further exacerbation of anemia during this period [42]. A decrease in circulating hemoglobin levels results in a reduction in oxygen availability for cells, which intensifies the hypoxia caused by COVID-19-induced acute respiratory distress syndrome [43]. In our study, anemia and hemoglobin counts had direct and inverse dependence, respectively, on the risk of ICU admissions of COVID-19-infected pregnant women. Nevertheless, none of these factors were highly associated with the risk of ICU admission for these patients.

“Loss of smell”, a common symptom in COVID-19 cases, has been found primarily in the less severe cases [44,45]. The mechanism that explains “loss of smell” in COVID-19 is largely unknown, so it is unclear whether these findings may contradict previous evidence.

Gestational age at infection ≥ 31 weeks was an independent risk factor for severe–critical COVID-19 [46,47,48]. This phenomenon has also been observed in influenza infection [49], probably because of the physiological changes during pregnancy [50], such as reduced respiratory capacity, vascular and hemodynamic changes like an increased body fluid in the third space, and compromised immune system due to the need for immune tolerance for the fetus, which develop as pregnancy advances [51]. There is, however, some controversy regarding the risk that gestational age represents for COVID-19-infected pregnant women [52,53], as not all studies have identified this association.

A bidirectional relationship between kidney function and COVID-19 disease has been suggested [54]. Patients with low renal function have an increased risk of critical COVID-19 [55] and a higher risk of ICU admission [56,57] or mortality [58], although none of these studies have exclusively considered pregnant women population. Furthermore, while chronic kidney disease is associated with impairment of the immune system, it is still unknown whether their worse COVID-19 outcome can be explained by a weaker antiviral response or by systemic inflammation [59].

Other studies have identified that more advanced age, due to possible age-related immune changes, potential comorbidities, and elevated BMI, represents risk factors for more severe COVID-19 infection in pregnant women [53]. Previous research found pre-pregnancy obesity to be a strong predictor of ICU admission, as it is linked with more severe COVID-19 cases, respiratory issues, and other metabolic complications [60]. Although we found this association in the unadjusted data, the final machine learning model did not include these variables as having a relevant effect.

A similar lack of effects in the final machine learning model occurs with comorbidities like preeclampsia, hypertension, hyperglycemia, or gestational diabetes [56]. Hypertension and other cardiovascular may elevate the likelihood of ICU admission for pregnant patients with COVID-19, as these conditions exacerbate the body’s inflammatory response, as well as diabetes, gestational diabetes, or preeclampsia. A possible explanation for the lack of effect of these problems is that they are actually reflected in the alterations in the inflammatory biomarkers (altered white blood cell count and elevated CRP) that, in this study, are actually associated with a higher risk of admission to ICU, since these are parameters that are also altered in these obstetric conditions.

Severe dyspnea, low oxygen saturation (<94%), and the need for mechanical ventilation are direct predictors of ICU care. Respiratory complications are often more severe in pregnant women due to reduced lung capacity as the pregnancy progresses and are linked to pneumonia and the need for ventilation. COVID-19-related pneumonia is a primary predictor of ICU admission, as it often requires mechanical ventilation to support oxygen levels [61].

Pregnant women experience unique physiological and immunological changes that differentiate their response to COVID-19 from the general population. These differences may impact the progression of the disease, clinical manifestations, management strategies, and outcomes. Pregnancy maintenance relies on finely tuned immune adaptations [57] as it must maintain tolerance to the fetus while preserving innate and adaptive immune mechanisms for protection against microbial challenges, exhibiting a shift to Th2-dominant immunity to protect the fetus, which may make them more vulnerable to viral infections, including COVID-19 [59]. CRP and other inflammatory biomarkers may be naturally elevated in pregnancy, complicating the interpretation of higher risk in pregnant women compared to the general population. These adaptations of active immunologic tolerance are precisely timed and reflect an immune clock of pregnancy in women delivering at term. The differences in susceptibility, variability in progression, and differences in the risk of COVID-19 infection severity are also associated with host genetic factors [59], like unfavorable genotypes of IFNL3/IFNL4 SNPs. Among pregnant patients with confirmed SARS-CoV-2 infection, reduced mucosal antibody responses were associated with greater infectious virus recovery and viral RNA levels [60].

Pregnancy is associated with several factors that mimic the symptoms of COVID-19, like increased blood volume, cardiac output, heart rate, shortness of breath, and fatigue, which may overlap with normal pregnancy, potentially delaying COVID-19 diagnosis. Normal changes in pregnancy are also linked to more risk of severe COVID-19 complications like reduced lung capacity due to diaphragm elevation and increased oxygen consumption or its hypercoagulable state, increasing the risk of thromboembolic events.

Our study has some limitations. It is a retrospective study that was conducted in two centers. Women included in this analysis were those who were hospitalized either through spontaneous demand or after being referred from ambulatory care, which could leave more vulnerable groups underrepresented. Because the participants were recruited from 2020 to 2021, they were unvaccinated, as vaccination was not authorized in Kazakhstan for pregnant women during that period. This period includes the second wave of COVID-19 cases in the country, which are mostly related to the Delta variant. This variant has been associated with more severe progression in pregnancy with increased ICU admissions and increased need for advanced oxygen support. The Delta variant is associated with more unfavorable maternal and neonatal outcomes, including preterm births, SGA infants, lower Apgar scores, higher maternal and fetal mortality, higher maternal admission to ICU, higher CRP levels, as well as PCT and IL-6, higher levels of lymphocytes, D-dimer, and transaminases. It is also associated with a higher rate of placental SARS-CoV-2 detection [62]. The possibility that other variants or vaccination status could alter the model’s outcomes could not be derived from these data and may limit the generalizability of these findings.

During this period, there were no homogeneous protocols/ICU admission criteria for these patients, and they were treated with different drugs (corticosteroids, lopinavir/ritonavir, azithromycin, hydroxychloroquine, interferon beta, tocilizumab, and prophylactic or therapeutic heparin). We could not analyze the possible association with higher risk of ICU admission of parameters like ferritin, interleukin-6, or d-dimer as they were only available for a reduced number of cases. Ferritin, interleukin-6, or d-dimer provide insight into the immunological and inflammatory pathways involved in severe COVID-19. Although their absence limits understanding of the disease’s immunological and inflammatory pathways involved in severe COVID-19 and may hinder the development of tailored management strategies, CRP is considered as the inflammatory biomarker that better mirrors the course of the disease compared to d-dimer or ferritin [63]. Excessive production of interleukin-6 is associated both with more severe COVID-19 cases [64] as well as with adverse pregnancy outcomes like preterm delivery, preterm premature rupture of the membranes, and chorioamnionitis [65]. Significantly higher interleukin-6 levels in pregnant women with no significant difference have been observed between the pregnancy trimesters [66].

This study also has some relevant strengths. First, the large number of cases was analyzed. Second, the methods that facilitate transparency and interpretability of the data were used, helping to explain not only what the model predicted but why. Third, early identifying women at higher risk of complications during pregnancy may also help to offer psychological support to couples in these stressful situations [67]. Finally, the methodological approach for this work may be extended in cases of other infectious diseases that may affect pregnancy outcomes [68] as well as in situations when excess demand requires valid triage methods to allocate patients to always limited resources, like ICU beds.

## 5. Conclusions

Routinely collected clinical and laboratory data of COVID-19-infected pregnant women may help recognize high-risk groups who are more liable for complications and more severe course or prognosis and require an ICU admission. Leucocyte counts, C-reactive protein, pregnancy week, eGFR, and hemoglobin appeared as significant predictors of high risk of severe infection requiring ICU admission. The predictive model may be an efficient support tool for prioritizing care of COVID-19-infected pregnant women in clinical practice [69]. These findings may also contribute to enhancing our understanding of the pathogenesis of the disease and subsequently improve the outcome of patients. In this work, the identified best predictive model, which was logistic regression with *L_2_* regularization, achieved an AUC of 0.845 and sensitivity and specificity of 0.600 and 0.896 on test data, respectively. This result showcases the potential usage of machine learning to serve as an efficient and supportive tool for prioritizing care of COVID-19-infected pregnant women in clinical practice, especially in resource-strained healthcare systems.

## Figures and Tables

**Table 1:** Baseline Demographic and Clinical Characteristics *n* = 1168. To characterize the difference between those who were and were not admitted to ICU, *p*-values of two-sided Welch’s *t*-test (for numerical features) and chi-square test (for categorical features) were calculated.

	Trimester of Gestation					
Characteristic	1st Trimester (*n* = 59)	2nd Trimester (*n* = 293)	3rd Trimester (*n* = 813)	ICU Admitted (*n* = 110)	Not Admitted to ICU (*n* = 1058)	*p*-Value
**Feature**						
Age	30.15 ± 5.92	30.13 ± 5.64	29.66 ± 5.61	31.52 ± 5.67	29.63 ± 5.59	0.0011
Blood type						0.3621
A	12	67	252	38	295	
AB	3	28	67	9	90	
B	15	95	225	33	300	
O	28	99	251	29	355	
Rh factor						0.9769
−	4	14	29	4	44	
+	54	275	765	105	995	
Days of admission after symptom onset	4.20 ± 2.44	4.41 ± 3.07	4.62 ± 3.63	5.83 ± 3.83	4.42 ± 3.38	0.0003
Length of hospital stay	7.64 ± 1.89	8.31 ± 3.36	6.71 ± 3.04	8.53 ± 5.52	7.02 ± 2.76	0.0057
**Obstetric history**						
Number of children	1.29 ± 1.34	1.17 ± 1.06	1.69 ± 1.35	1.98 ± 1.26	1.50 ± 1.30	0.0002
Number of pregnancies	2.68 ± 1.71	2.66 ± 1.60	2.79 ± 1.69	2.95 ± 1.76	2.73 ± 1.66	0.2085
Number of deliveries	1.31 ± 1.36	1.20 ± 1.06	1.71 ± 1.36	2.02 ± 1.23	1.52 ± 1.31	<0.001
Multiple gestations	0	4	4	1	7	
**Laboratory tests**						
Hemoglobin	120.19 ± 19.58	105.79 ± 11.34	105.67 ± 14.79	97.97 ± 16.08	107.36 ± 14.2	<0.001
Leucocytes	6.91 ± 2.62	8.25 ± 3.38	9.33 ± 3.65	12.58 ± 4.93	8.56 ± 3.20	<0.001
Neutrophils	73.06 ± 9.88	79.50 ± 8.74	79.89 ± 10.3	86.30 ± 17.00	78.75 ± 8.70	<0.001
Lymphocytes	24.50 ± 13.63	16.95 ± 9.11	16.81 ± 8.27	12.40 ± 8.29	17.73 ± 8.92	<0.001
Platelets	205.95 ± 68.32	210.81 ± 77.57	217.69 ± 85.43	250.09 ± 134.92	211.86 ± 74.40	0.0043
APTT	29.08 ± 3.19	31.37 ± 11.08	31.93 ± 13.62	35.69 ± 13.49	31.22 ± 12.51	0.0012
ALT	29.23 ± 31.32	37.80 ± 58.26	23.46 ± 34.89	35.64 ± 83.34	26.65 ± 35.75	0.2808
ACT	27.47 ± 17.19	35.88 ± 42.0	29.69 ± 25.48	41.71 ± 59.44	30.10 ± 25.51	0.0509
**Comorbidities and complications**						
Preeclampsia	0	3	23	10	16	<0.001
Small for gestational age	0	1	18	2	17	1.0
Intrauterine growth restriction	0	0	17	3	14	0.4521
Hypertension	1	17	67	23	63	<0.001
Hyperglycemia	3	53	121	29	148	<0.001
Gestational diabetes	1	11	33	13	32	<0.001
Anaemia	13	162	599	90	685	<0.001
Pneumonia	26	152	437	68	549	0.05
**Clinical symptoms and severity of COVID-19**						
Fever	34	125	231	31	359	0.2666
Cough	52	263	619	78	857	0.0156
Weakness	57	280	726	98	967	0.5249
Sore throat	31	191	494	55	662	0.0133
Shortness of breath	14	59	149	30	192	0.0282
Myalgia	15	95	217	33	294	0.7038
Loss of smell and/or taste	35	104	208	21	326	0.0143
Runny nose	46	230	613	75	816	0.0475
Diarrhea	6	11	13	0	30	0.1409
Chest discomfort	11	60	145	17	199	0.4633
Sweating	3	5	19	3	24	1.0
ICU Admission	0	13	96			

**Table 2:** A summary of the hyperparameter spaces.

Classifier	Hyperparameter	Hyperparameter Space
LR	penalty	*L_2_*
	regularization parameter	100, 10, 1.0, 0.1, 0.01
RF	number of estimators	1, 2, 5, 10
	maximum depth	2, 5, 10
	maximum features	‘sqrt’, ‘log2’
LDA	solver	‘svd’, ‘lsqr’, ‘eigen’
	tolerance	0.00001, 0.0001, 0.0003
GB	number of estimators	10, 20, 30
	learning rate	0.001, 0.01, 0.1
XGB	maximum depth	5, 10
	number of estimators	10, 20, 30
	learning rate	0.001, 0.01, 0.1
ADB	number of estimators	10, 20, 30
	learning rate	0.001, 0.01, 0.1
	algorithm	‘SAMME’, ‘SAMME.R’
GNB	-	-
KNN	number of neighbors	3, 5

**Table 3:** The *F*_1_-scores, decision-making threshold value, and selected hyperparameter spaces of classifiers during the model selection. The best model is identified in bold.

Classifier	*F*_1_-Score	Threshold	Hyperparameter Space
**LR**	**0.49 ± 0.04**	**0.18**	**regularization parameter: 0.01**
RF	0.48 ± 0.12	0.18	maximum depth: 2; maximum features: log2; number of estimators: 10
LDA	0.47 ± 0.03	0.20	solver: “svd”; tolerance: 0.00001
GB	0.47 ± 0.11	0.26	learning rate: 0.1; number of estimators: 20
XGB	0.45 ± 0.08	0.26	learning rate: 0.1; number of estimators: 30; maximum depth: 10
ADB	0.42 ± 0.05	0.34	algorithm: “SAMME.R”; learning rate: 0.1; number of estimators: 30
GNB	0.31 ± 0.07	0.71	-
KNN	0.31 ± 0.04	0.00	number of neighbors: 5

**Table 4:** Test-set performance metrics estimated using the best predictive model (logistic regression).

Accuracy	Precision	Sensitivity	Specificity	G-Mean	*F*_1_-Score	ROC AUC
0.866	0.389	0.600	0.896	0.733	0.472	0.845

**Table 5:** Confusion matrix obtained on the test set using the best predictive model (logistic regression). Positive and negative labels represent ICU admitted and non-admitted, respectively.

	Predicted		
**Actual**		Negative	Positive
	Negative	True Negative: 283	False Positive: 33
	Positive	False Negative: 14	True Positive: 21

## Data Availability

Data are available from the correspondence author upon request due to privacy and ethical restrictions.

## Supplementary Materials

The following supporting information can be downloaded at: https://www.mdpi.com/article/10.3390/jcm13247705/s1, Table S1: Description of features.
