# Supplementary material for: Predicting Intensive Care Unit Admission in COVID-19-Infected Pregnant Women Using Machine Learning
Source: J Clin Med. 2024 Dec 17;13(24):7705. doi: 10.3390/jcm13247705 (PMC11677355; doi:10.3390/jcm13247705)
Supplement: Supplementary file 1 [file jcm-13-07705-s001.zip › jcm-3320369-supplementary.pdf]

Table S1 Description of features

Feature	Data type
Age	numerical
Blood type	categorical
Rh factor	binary
Days of admission after symptoms onset	numerical
Length of hospital stay	numerical
**Obstetric history**	
Pregnancy week	numerical
Number of children	numerical
Number of pregnancies	numerical
Number of deliveries	numerical
Multiple gestations	binary
**Laboratory tests**	
Haemoglobin	numerical
Leucocytes	numerical
Neutrophils	numerical
Lymphocytes	numerical
Platelets	numerical
APTT	numerical
ALT	numerical
ACT	numerical
Total bilirubin	numerical
CRP	numerical
Creatinine	numerical
Glucose	numerical
eGFR	numerical
**Comorbidities and complications**	
Preeclampsia	binary
Small for gestational Age	binary
Intrauterine growth restriction	binary
Hypertension	binary
Hyperglycaemia	binary
Gestational diabetes	binary
Anaemia	binary
Hypotension	binary
Pneumonia	binary
**Clinical symptoms and severity of COVID-19**	
COVID-19 Severity	categorical
Fever	binary
Cough	binary
Weakness	binary
Sore throat	binary
Shortness of breath	binary
Myalgia	binary
Loss of smell and/or taste	binary
Runny nose	binary
Diarrhoea	binary
Chest discomfort	binary
Sweating	binary
Heartbeat	numerical
Saturation	numerical

ALPTT - Activated Partial Thromboplastin Time
ALT - Alanine Aminotransferase
eGFR - estimated Glomerular Filtration Rate
CRP - C-Reactive Protein
ACT - Activated Clotting Time
SGA - Small for gestational age
